# Isolation and identification of a new biocontrol bacteria against *Salvia miltiorrhiza* root rot and optimization of culture conditions for antifungal substance production using response surface methodology

**DOI:** 10.1186/s12866-022-02628-5

**Published:** 2022-09-30

**Authors:** Rongbo Sa, Song He, Dongdong Han, Mengjiao Liu, Yunxia Yu, Rongen Shang, Meimei Song

**Affiliations:** Shandong First Medical University & Shandong Academy of Medical Sciences, Taian, China

**Keywords:** *Salvia miltiorrhiza* root rot, Biocontrol bacteria, Isolation and identification, Antifungal substance, Optimization, Response surface methodology

## Abstract

**Background:**

*S. miltiorrhiza* root rot is a soil-borne disease mainly caused by *Fusarium solani* and *Fusarium oxysporum*, which has spread rapidly in China in recent years. To reduce the amount of pesticides to control this plant fungal disease, biological control using endophytic bacteria is a promising method. Many endophytic bacteria show good biocontrol potential against various plant fungal diseases. The aims of this study were to isolate and identify endophytic bacteria with antifungal activity from *Salvia miltiorrhiza* plant tissue. In order to increase antifungal substances production, the culture conditions of the isolated DS-R5 strain were optimized through response surface methodology.

**Results:**

Thirteen endophytic bacteria with antifungal activity against the target pathogenic fungus were successfully screened. The DS-R5 strain that had the strongest antifungal activity was identified based on morphological, physiological and biochemical characteristics, 16S rRNA and *gyrB* sequence analysis.The results of response surface methodology experiments showed that the optimal values of the three significant factors were as follows: medium volume, 51.0 ml; initial pH, 6.7; fermentation temperature, 33.1 °C. Under these optimal culture conditions, the titer of antifungal substances produced by the DS-R5 strain was 77.6% higher than that under the initial culture conditions.

**Conclusions:**

The antifungal activity of endophytic bacteria from *Salvia miltiorrhiza* has been demonstrated for the first time, which may benefit future crop quality and production. In addition, response surface methodology can be well applied the optimization of culture conditions for antifungal substance, which lays the foundation for further research on strain DS-R5.

## Introduction

*Salvia miltiorrhiza* is a perennial herbal medicinal plant whose root and rhizome is used as medicine to remove blood stasis and relieve pain, promote blood circulation and regulate menstruation, nourish the heart and reduce anxiety. It is also widely used in the treatment of cardiovascular and cerebrovascular diseases [1–3]. Under artificial cultivation conditions, due to poor diversity and low richness of the biological communities in the planting environment, serious diseases and pests often occur, which affect the growth and development of *S. miltiorrhiza* and reduce yield and quality of the medicinal material [4]. *S. miltiorrhiza* root rot is a soil-borne disease, mainly caused by *Fusarium solani* and *Fusarium oxysporum*, that has spread rapidly in China in recent years [5]. After becoming infected, *S. miltiorrhiza* plants grow weak, and the root xylem completely rots to become black and brown, which affects the appearance, character and quality to the point that they do not meet the requirements for medicinal use. At present, the main control method for fungal diseases of medicinal plants is chemical control. Although chemical control can effectively inhibit the occurrence of fungal diseases in the short term, the application of chemical agents greatly increases pesticide residues in the soil, pollutes the environment and enhances the disease resistance of pathogenic microorganisms [6]. In recent years, more and more studies have shown that biological control using endophytes is an important alternative to chemical control [7–9].

Endophytic bacteria live in healthy plant tissues without causing significant damage to the plant and can improve the resistance of plants to biotic or abiotic stresses, making them a good choice as potential biocontrol bacteria [10]. People have been looking for endophytic bacteria for biocontrol strains against many plant pathogens for decades, and many endophytic bacteria show good biocontrol potential against various plant fungal diseases [11]. *Paenibacillus polymyxa* is a gram-positive spore-producing bacteria formerly known as *Bacillus polymyxa* that is a good target for screening biocontrol bacteria of plant diseases [12]. To control many different plant diseases, *P. polymyxa* releases many different antimicrobial components, including proteins, phenols, peptides, pyrazines and nucleosides [13]. As *P. polymyxa* can be used as a biological pesticide to control a variety of plant diseases and it is safe to use without environmental pollution, *P. polymyxa* has been designated as one of the commercially available microorganisms by the U.S. Environmental Protection Agency [14].

In the process of microbial fermentation, the environmental conditions need to be strictly controlled in order to improve the yield of fermentation products [15]. To date, optimization of culture conditions is still the most important method to improve fermentation efficiency. Therefore, the optimization of culture conditions for the production of antimicrobial substances is of great significance. At present, one of the main problems to be solved in the application of biological control agents is that biocontrol bacteria secrete less effective antifungal substances during the fermentation process [16]. In order to maximize antifungal activity, the fermentation medium and culture conditions for antifungal substances production by the DS-R5 strain must be optimized. In our previous study, the fermentation medium for antifungal substances production by the DS-R5 strain was optimized using one-factor-at-a-time, Plackett–Burman (PB) design and Box-Behnken design experiments. Increasingly, researchers have shown that environmental conditions such as culture time, shaker speed, temperature, pH and inoculum percentage play a significant role in inducing antimicrobial substance synthesis [17, 18].

In this study, the endophytic strain DS-R5 with strong antifungal activity against *F. solani* was isolated from *S*. *miltiorrhiza* root tissue and identified as *P*. *polymyxa* using morphology and molecular biology. The results of pot experiments showed that the control efficacy of the DS-R5 strain on *S*. *miltiorrhiza* root rot was 61.4%. Therefore, the DS-R5 strain has good biocontrol potential for preventing and controlling *S. miltiorrhiza* root rot and offers the prospect of further development. In addition, response surface methodology (RSM) was used to optimize the culture conditions for antifungal substances synthesis in order to maximize their production. Our objectives in studying the specific components of active antifungal substances of the DS-R5 strain are to clarify its antifungal mechanism and to assess its potential as a biocontrol agent for preventing and controlling *S. miltiorrhiza* root rot.

To the best of our knowledge, we have isolated and identified a biocontrol bacterium from the medicinal plant *S. miltiorrhiza* for the first time in this study and demonstrated its antagonistic effects on *F. solani*, the pathogen of *S. miltiorrhiza* root rot. The results of this research demonstrate the potential of the DS-R5 strain as a biocontrol agent for preventing and controlling *S. miltiorrhiza* root rot. In addition, this study provides a reference for the optimization of culture conditions and lay a theoretical foundation for the preparation of biocontrol agents by the DS-R5 strain.

## Materials and methods

### Isolation of endophytic antagonistic bacteria and growth conditions

Healthy *S. miltiorrhiza* plants with good growth were collected and cleaned with water. The material surface was disinfected by soaking in 75% ethanol for 1 min and rinsing with sterile water three times, then soaking in 0.1% mercuric chloride for 3 min and rinsing with sterile water three times. The treated *S. miltiorrhiza* tissues, including the root, stem and leaf, were ground into a pulp with a mortar on a sterile operating table, diluted 10 times in sterile water, coated on LB medium and cultured at 37 °C for 3 days. When visible colonies appeared on the culture medium, the colonies were picked and pure cultures were obtained by repeated streak-line separation. Sterile water was used to take the last rinse of *S. miltiorrhiza* tissues and coat it on LB medium. No colonies grew after incubation at 37 °C for 3 days, indicating that the sample surface was thoroughly disinfected.

The fermentation medium for antifungal substances contained the following components: 15.0 g/l sucrose, 30 g/l soluble starch, 7.0 g/l corn steep liquor, 10.0 g/l (NH_4_)_2_SO_4_, and 0.7 g/l KH_2_PO_4_.

The DS-R5 strain was inoculated into Luria–Bertani (LB) medium and cultured at 37 °C in a rotary shaker (KS4000i, IKA, German) at 200 rpm for 16 h to prepare the inoculum. A 2% (v/v) inoculum was inoculated into a flask (250 ml) containing 100 ml fermentation medium and incubated at 37 °C in a rotary shaker at 200 rpm for 72 h to produce antifungal substances.

### Sample collection and pathogenic fungi

Healthy 3-year-old *S. miltiorrhiza* plants were collected from an *S. miltiorrhiza* plantation at the foot of Mount Tai, China. During sampling, the entire plant was pulled out with its roots, placed in a sterile bag and brought back to the laboratory for endophytic bacteria isolation.

The tested pathogenic fungi used in this study, *F*. *solani*, *Rhizoctonia solani*, *Alternaria alternate*, *Fusarium oxysporum*, *Colletotrichum orbiculare*, *Fusarium graminearum*, *Botryosphaeria ribis* and *Fusarium pseudograminearum,* are all preserved in our laboratory.

### Crude extract preparation of antifungal substances produced by the DS-R5 strain

After fermentation, the supernatant of the fermentation broth was adjusted to pH 2.0 with 6 mol/l hydrochloric acid, then centrifuged at 6000 rpm for 10 min after overnight storage at 4 °C. The precipitate was then lyophilized to obtain a light yellow powder, which was the crude extract of antifungal substances used to make the standard curve for titer determination.

### Titer determination of the DS-R5 strain fermentation broth

A crude extract solution of antifungal substances with a concentration of 10,000 mg/l was prepared with 70% methanol solution, then diluted into 5000, 2500, 1250, 625 and 312.5 mg/l solutions. After titer determination, the medium was melted and cooled to 50–55 °C, and the spore suspension of *F. oxysporum* at a concentration of approximately 1 × 10^5^ colony forming units/ml was added at a ratio of 0.5% (v/v) to prepare mixed spore plates. Sterilized Oxford disks were placed on the mixed spore plates, and 200 µl of antifungal substance solution was added at various concentrations. After culture at 30 °C for 48 h, the diameter of the inhibition zone were measured using the cross method to create a standard curve for the titer bioassay. The titer of the fermentation broth was then calculated according to the following validated standard equation: y = 8.7619x-6.4762 (*R*^2^ = 0.9911), where y is the diameter of the inhibition zone (mm) and x is the logarithmic value of the concentration of the antifungal substance solution.

### Screening of antagonistic bacteria by plate confrontation assay

According to the method reported by Khalil et al. [19], the endophytic antagonistic bacteria isolated from *S. miltiorrhiza* were screened for their antagonistic activity against the target pathogen *F. oxysporum* using the dual plate confrontation assay method. A 5-mm puncher was used to punch holes on *F. oxysporum* plates cultured for 7 days. The 5-mm fungal cake was inoculated in the center of a potato dextrose agar (PDA) plate, and the isolated endophytic bacteria were inoculated in parallel lines 2.5 cm away from the fungal cake. A fungal plate without antagonistic bacteria was used as the control and placed in a 30 °C incubator for constant temperature culture. When the pathogen colonies in the control group were overgrown on the bottom of the dish, the width of the inhibition zone of the treatment group was measured. Each treatment was repeated three times.

### Identification of endophytic antagonistic bacteria

#### Morphological, physiological and biochemical identification

The DS-R5 strain was inoculated onto LB plates and incubated in a 37 °C incubator (CT-260R, BOLV, China) for 24 h. The morphological characteristics of colonies were observed and conventional Gram staining was carried out. The morphology of bacterial cells was observed using an optical microscope (CX-31, Olympus, Japan) and a scanning electron microscope (S-3400 N, Hitachi, Japan). Physiological and biochemical identification of the DS-R5 strain was carried out according to Bergey’s Manual of Systematic Bacteriology (second edition, 2004).

#### Molecular identification

Single bacterial colonies were selected and cultured in liquid LB medium at 30 °C with shaking at 160 rpm for 14–18 h to obtain a bacterial suspension. Genomic DNA was extracted using a bacterial genomic DNA extraction kit.

Amplification of the 16S rRNA gene: The 16S rRNA gene sequence was amplified by polymerase chain reaction (PCR) according to the protocol of Aravind et al. [20]. Using total DNA as the template, the 16S rRNA sequence of the DS-R5 strain was amplified with universal primers 27F (5′-AGAGTTTGATCCTGGCTCAG-3′) and 1492R (5′-GGTTACCTTGTTACGACT-3′). The PCR reaction contained the following: 12.5 μl 2 × Taq PCR Master Mix, 1.0 μl upstream and downstream primers (10 μmol/l), 0.5 μl DNA template and 10.0 μl ddH_2_O. The PCR reaction conditions were as follows: 95 °C for 5 min; 30 cycles of 94 °C for 1 min, 55–58 °C for 1 min and 72 °C for 90 s; and 72 °C for 10 min.

Amplification of the *gyrB* gene: The *gyrB* gene exists in most bacteria and, because it does not undergo frequent horizontal transfer, can be used to effectively distinguish between closely related species [21]. In order to clarify the species of the DS-R5 strain, *gyrB* gene cloning and sequencing were used for its identification. The primer pair *gyrB-*F (5′-GAAGTCATCATGACCGTTCTGCAYGCNGGNGGNAARTTYGA-3′) and *gyrB*-R (5′-AGCAGGGTACGGATGTGCGAGCCRTCNACRTCNGCRTCNGTCAT-3′) was used for amplification of the *gyrB* gene [22]. The PCR reaction contained the following: 12.5 μl 2 × Taq PCR Master Mix, 0.5 μl upstream and downstream primers (10 μmol/l), 1.0 μl DNA template and 10.5 μl ddH_2_O. The PCR reaction conditions were as follows: 94 °C for 5 min; 35 cycles at 94 °C for 30 s, 55–58 °C for 30 s and 72 °C for 1 min; and 72 °C for 10 min.

PCR products of the 16S rRNA and *gyrB* genes were sequenced by Qingdao Yixin Testing Technology Co., Ltd (Qingdao, China). BLAST comparisons were performed on the National Center for Biotechnology Information (NCBI) to select strains with high similarity. The neighbor-joining method was used to construct a phylogenetic tree in MEGA 7.0 software.

### Determination of the antimicrobial spectrum of the DS-R5 strain

Antagonistic experiments were conducted with the DS-R5 strain against eight common pathogenic plant fungi. The tested fungi were prepared into a cake with a diameter of 5 mm and placed in the center of a PDA plate. Then, the DS-R5 strain was spread 2.5 cm away from the test pathogen cake. After 7 days of constant temperature culture at 30 °C, the width of the inhibition zone was determined.

### Pot experiments of the DS-R5 strain against *S. miltiorrhiza* root rot

Sixty potted *S. miltiorrhiza* seedlings with good growth were selected and divided into three groups: a healthy control group (no inoculation), a pathogenic control group (inoculation with pathogenic fungi) and a treatment group (inoculation with pathogenic fungi and antagonistic bacteria), with 20 plants in each group. The pathogenic control group and the treatment group were irrigated with a 20-ml *F. solani* spore suspension with a concentration of 1 × 10^6^ spores/ml. After 10 days, the roots of the pathogenic control group and the treatment group were irrigated with 20 ml DS-R5 strain fermentation broth. The group inoculated with 20 ml uninoculated sterile medium was used as a healthy control group. After 60 days, the control effect of the DS-R5 strain on *S. miltiorrhiza* root rot was analyzed statistically. According to the lesion area on the root tissue of *S. miltiorrhiza* seedlings, the disease was divided into five grades: grade 1, no lesions; grade 2, lesion area < 5%; grade 3, lesion area of 5–20%; grade 4, lesion area of 21 repeated streaked separation 50%; and grade 5, lesion area > 50%. The disease index was calculated as follows: disease index = Σ (disease grade × number of plants of this disease grade) / (maximum disease grade × total number of plants) × 100. The control efficacy was calculated as follows: control efficacy (%) = (disease index of control group—disease index of treatment group) / control disease index × 100.

### Analytical methods for optimizing culture conditions

#### One‑factor‑at‑a‑time experimental design

The main factors affecting antifungal substance synthesis are medium volume, initial pH, inoculum size, fermentation time, rotary speed and fermentation temperature. Considering the culture conditions reported in previous studies, the ranges of values of the main factors in this study were set as follows: medium volume of 30, 60, 90, 120, 150 and 180 ml in 250 ml shaking flasks; initial pH of 4.0, 5.0, 6.0, 7.0, 8.0 and 9.0; inoculum size of 0.5, 1.0, 1.5, 2.0, 2.5 and 3.0%; fermentation time of 3, 4, 5, 6, 7 and 8 days; rotary speed of 0, 50, 100, 150, 200 and 250 rpm; and fermentation temperature of 20, 25, 30, 35, 40 and 45 °C.

#### RSM experimental design

##### **PB design**

The PB design is a two-level experimental design method that screens for important factors using the least number of experiments to accurately estimate the effect values of different factors [23]. Based on the data from one‑factor‑at‑a‑time experiments, a PB design with a set of 12 experiments was used to identify the significant factors affecting antifungal substances production, with the response value as the titer of the antifungal substances. The six parameters, A, B, C, D, E and F, represent medium volume, initial pH, inoculum size, fermentation time, rotary speed and fermentation temperature, respectively. The high (+ 1) and low (-1) levels of these factors are shown in Table 1, with the high level being approximately 1.5 times that of the low level. Regression analysis was carried out using Minitab 17.0 software (Minitab, LLC, State College, PA, USA) to assess statistical significance.

**Table 1:** Factors and levels of PB design

Factors	Level	
	-1	+ 1
A: medium volume (ml)	50	70
B: initial pH	5.0	7.0
C: inoculum size (%)	1.5	2.5
D: fermentation time (d)	6.0	8.0
E: rotary speed (rpm)	120	180
F: fermentation temperature (°C)	25	35

##### **Steepest ascent design**

After significant influencing factors were screened from PB design results, the gradient direction of the response value was taken as the climbing direction. According to the effect value of each factor, the change step size was determined to design steepest ascent experiments so that the response value quickly approached the maximum response region.

##### Central composite design (CCD)

CCD experiments were carried out using the three most important factors identified in PB experiments while fixing other non-critical factors. The factors and levels of CCD are shown in Table 2. The experimental data were fitted by quadratic regression to obtain a quadratic equation with interaction terms and square terms, and the main effect and interaction effect of each factor were analyzed. Finally, the optimal value was obtained within a certain level range. The commonly used second-order equation model of response surface analysis is as follows:

**Table 2:** Factors and levels of CCD

Factors	Level				
	-1.682	-1	0	+ 1	+ 1.682
A: medium volume (ml)	43	50	60	70	77
B: initial pH	4.3	5.0	6.0	7.0	7.7
F: fermentation temperature (°C)	21.6	25	30	35	38.4

1 $$Y= {\alpha }_{0}+ {\alpha }_{1}{X}_{1}+{\alpha }_{2}{X}_{2}+{\alpha }_{3}{X}_{3}+{\alpha }_{12}{X}_{1}{X}_{2}+{\alpha }_{13}{X}_{1}{X}_{3}+{\alpha }_{23}{X}_{2}{X}_{3}+{\alpha }_{11}{X}_{1}^{2}+{\alpha }_{22}{X}_{2}^{2}+{\alpha }_{33}{X}_{3}^{2}$$

where *Y* refers to the response value, *X*_1_, *X*_2_ and *X*_3_ are the three most significant parameters identified by the PB design; *α*_0_ refers to the coefficient of the model; *α*_1,_ *α*_2_ and *α*_3_ refer to the linear correlation coefficient; *α*_12,_ *α*_13_ and *α*_23_ refer to the interaction coefficients of factors and *α*_11_; and *α*_22_ and *α*_33_ refer to the square coefficients of each factor.

##### **Verification experiments**

By fitting the experimental results of the optimization experiments with the RSM, each coefficient in the model was obtained by regression, and the extreme point and the value of the corresponding independent variable was determined by mathematical analysis of the multivariate function. Experiments were carried out according to the calculated parameters to verify the reliability of the model and determine the final optimization results.

## Statistics and data analysis

The data obtained from experiments were processed using SPSS 19.0 statistical software (IBM Corp., Armonk, NY, USA), and Duncan’s new multiple range method was used to analyze the significance of differences between treatments.

## Results

### Isolation and screening of endophytic antagonistic bacteria from *S. miltiorrhiza*

A total of 72 strains of bacteria were isolated from the roots, stems and leaves of *S. miltiorrhiza*. Among them, 39 strains were isolated from the root tissue, accounting for 54.2% of the total isolated bacteria, and 24 and 9 strains were isolated from the stems and leaves, accounting for 33.3% and 12.5% of the total isolated bacteria, respectively. Confrontation tests showed that 13 of the 72 endophytic bacteria had varying degrees of antagonistic effects against the target pathogenic fungus *F. solani* (Table 3). Among them, the DS-R5 strain had the strongest inhibitory effect, with an inhibitory zone width of 20.5 mm. Therefore, the DS-R5 strain was chosen for further research.

**Table 3:** Antagonistic effect of endophytic bacteria from *S*. *miltiorrhiza* against *F*. *solani*

Source of strain	Strain code	Width of inhibitory zone (mm)
	DS-R5	20.5 ± 1.9^a^
	DS-R12	13.4 ± 1.4^d^
Root	DS-R18	16.8 ± 1.1^b^
	DS-R25	9.2 ± 0.7^e^
	DS-R29	9.5 ± 0.9^e^
	DS-R31	15.9 ± 2.5^c^
	DS-S4 DS-S6 DS-S7 DS-S11 DS-S14	14.7 ± 1.2^b^ 18.1 ± 2.0^b^ 7.4 ± 1.1^j^ 7.9 ± 1.3^i^ 12.6 ± 1.7^ g^
	DS-S6 DS-S7	18.1 ± 2.0^a^ 7.4 ± 1.1^j^
Stem	DS-S7	7.4 ± 1.1^d^
	DS-S11	7.9 ± 1.3^d^ 12.6 ± 1.7^ g^
	DS-S14	12.6 ± 1.7^c^
Leaf	DS-L3 DS-L5	14.8 ± 2.3^a^
	DS-L5	12.1 ± 1.9^b^

*Note*: Data are mean ± standard deviation. Different lowercase letters in the same column indicate significant differences at the *P* < 0.05 level by Duncan’s new multiple range test

### Identification of the DS-R5 strain

#### Morphological characteristics

**Fig. 1:**
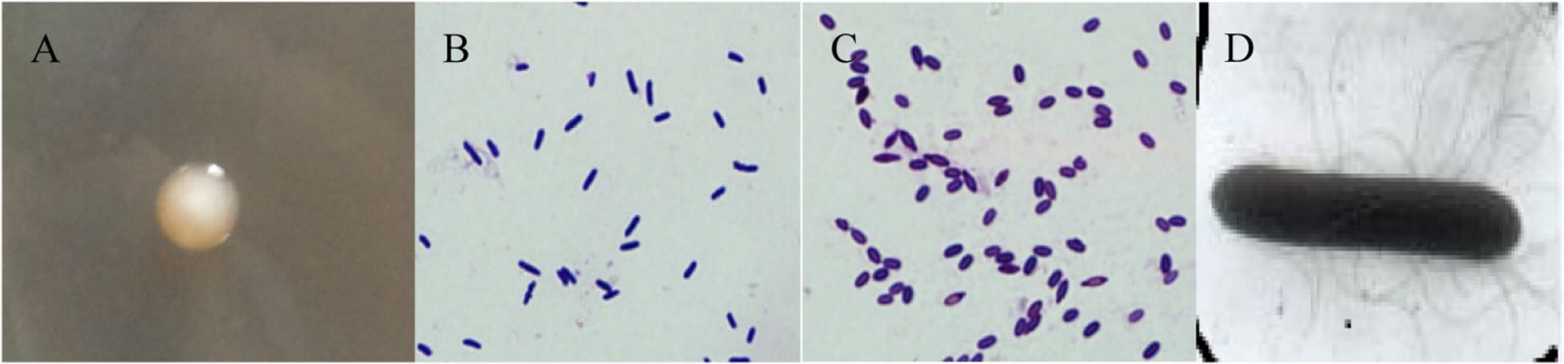
Morphological characteristics of the DS-R5 strain

After the DS-R5 strain was cultured on LB medium for 36 h, the colonies were smooth, wet, round in shape and not easily agitated (Fig. 1A). Under the light microscope, the bacteria cells were rod-shaped and gram-positive with oval spores growing in the middle (Fig. 1B, C). Scanning electron microscopy showed that the size of the bacterial cells was about 0.8 µm × 2.8 µm and that there were peripheral flagella (Fig. 1D). Some physiological and biochemical characteristics of the DS-R5 strain are shown in Table 4. The DS-R5 strain can use glucose and glycerol to produce acid. Starch hydrolysis, gelatin hydrolysis, anaerobic growth, contact enzyme, nitrate, Voges-Proskauer (V-P) and hydrolyzed casein reactions were positive. Oxidase, citrate, succinate and H_2_S production reactions were negative. The strain did not grow on medium containing 5% NaCl. Overall, the experimental physiological and biochemical parameters of the DS-R5 strain were consistent with *P. polymyxa* [24].

**Table 4:** Physiological and biochemical properties of the DS-R5 strain

Property	Result	Property	Result
Glucose	+	Amylo hydrolysis	+
Glycerol	+	Catalase	+
Oxidase	-	Casein hydrolysis	+
Succinate	-	V-P reaction	+
Anaerobic	+	Citrate utilization uuuutilization	-
Nitrate-reducing	+	H_2_S production	-
Isinglass Hydrolysis	+	Tolerance to NaCl	< 5%

Note: “ + ” positive; “-” negative

#### Molecular identification of the DS-R5 strain

**Fig. 2:**
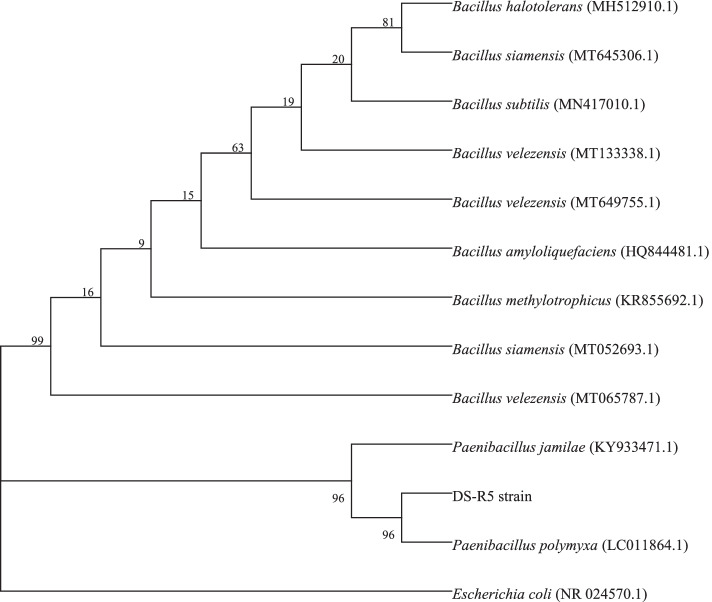
The phylogenetic tree of the DS-R5 strain based on 16S rRNA gene sequencing

**Fig. 3:**
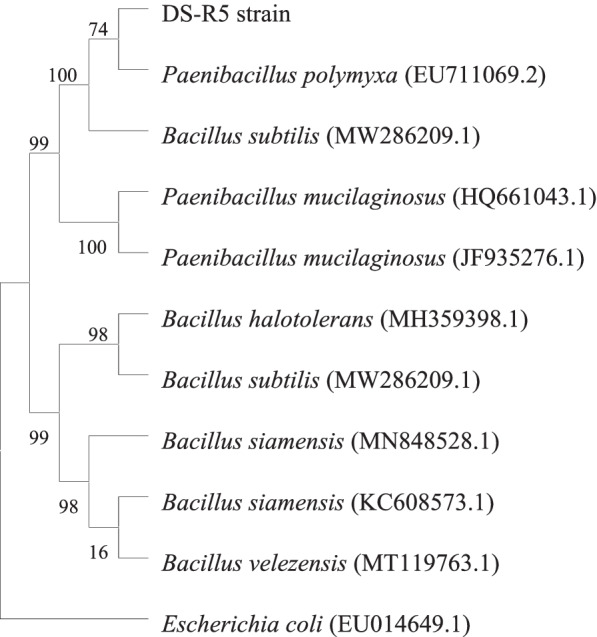
The phylogenetic tree of the DS-R5 strain based on *gyrB* gene sequencing

The 16S rRNA gene sequence of the DS-R5 strain was 1517 bp in length. After the sequence was submitted to NCBI, it was found by BLAST comparison that the 16S rRNA gene sequence of the DS-R5 strain was more than 99% similar to the gene sequences of *P*. *polymyxa*, *Paenibacillus mucilaginosus*, and *B. subtilis*. The phylogenetic tree showed that the DS-R5 strain and *P*. *polymyxa* belonged to the same branch with a support rate as high as 96% (Fig. 2). The phylogenetic tree based on the *gyrB* gene sequence showed that the DS-R5 strain and *P*. *polymyxa* belonged to the same branch and that the support rate was high (Fig. 3). Therefore, the DS-R5 strain was preliminarily identified as *P*. *polymyxa*. The combined morphological, physiological and biochemical characteristics and the results of gene sequence analysis of the 16S rRNA and *gyrB* genes confirmed that the DS-R5 strain was *P*. *polymyxa*.

#### Antimicrobial spectrum of the DS-R5 strain

The DS-R5 strain had different degrees of inhibitory effects on eight common pathogenic plant fungi (Table 5). Its antifungal activity against *R. solani* and *F. solani* was strongest, with inhibition zone widths of 21.2 mm and 20.5 mm, respectively. Its antifungal activity against *F. graminearum* and *F. pseudograminearum* was weak, with inhibition zone widths of 7.9 and 6.5 mm, respectively. Tests showed that the DS-R5 strain had a broad antimicrobial spectrum.

**Table 5:** Inhibitory effects of the DS-R5 strain on eight plant pathogens

Pathogenic fungi	Width of inhibitory zone (mm)	Pathogenic fungi	Width of inhibitory zone (mm)
*F*. *solani*	20.5 ± 1.9 b	*F*. *oxysporum*	17.8 ± 2.6 c
*R*. *solani*	21.2 ± 3.1 a	*F*. *pseudograminearum*	6.5 ± 0.8 g
*A*. *alternata*	15.9 ± 2.1 d	*C*. *orbiculare*	13.1 ± 1.7 e
*F*. *graminearum*	7.9 ± 1.1 f	*B*. *ribis*	12.8 ± 1.3 e

*Note*: Data are mean ± standard deviation. Different lowercase letters indicate significant differences at the *P* < 0.05 level by Duncan’s new multiple range test

### Control efficacy of the DS-R5 strain against *S. miltiorrhiza* root rot

The results of pot experiments showed that the disease index of the healthy control group without inoculation was 6.7, the disease index of the pathogenic control group inoculated with pathogenic fungi was 60.5 and the disease index of the treatment group inoculated with pathogenic fungi and the DS-R5 strain was 26.3. These results show that inoculation with the antagonistic strain DS-R5 had a significant effect on *S. miltiorrhiza* root rot, with a control efficacy of 61.4% in the treatment group (Table 6).

**Table 6:** Control efficacy of the DS-R5 strain against *S*. *miltiorrhiza* root rot

Treatment	Disease index	Control efficacy (%)
Healthy control group	6.7 ± 1.2 c	-
Pathogenic control group	60.5 ± 7.2 a	-
Treatment group	26.3 ± 5. 9b	61.4 ± 8.1

*Note*: Different lowercase letters in the same column indicate significant differences at the *P* < 0.05 level by Duncan’s new multiple range test

### Analytical results for fermentation condition optimization

#### Standard curve for antifungal substance bioassays

**Fig. 4:**
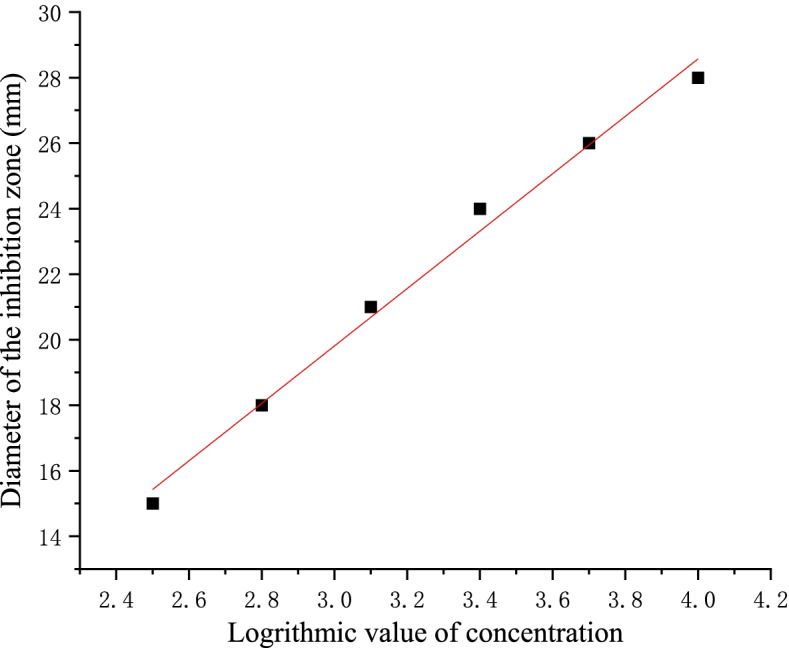
Standard curve for measuring antifungal substance titer

Bioassays were performed on a standard solution at five concentrations (10,000, 5000, 2500, 1250, 625, and 312.5 mg/l/), and the diameter of the zone of inhibition was recorded. Then, a standard curve was made with the logarithm of the concentration as the horizontal coordinate and the diameter of the zone of inhibition as the vertical coordinate (Fig. 4). It can be seen from the figure that the logarithm of the concentration had a linear relationship with the diameter of the inhibition zone, with a determination coefficient R^2^ = 0.9911, which meets the determination requirements.

#### One‑factor‑at‑a‑time experimental design

**Fig. 5:**
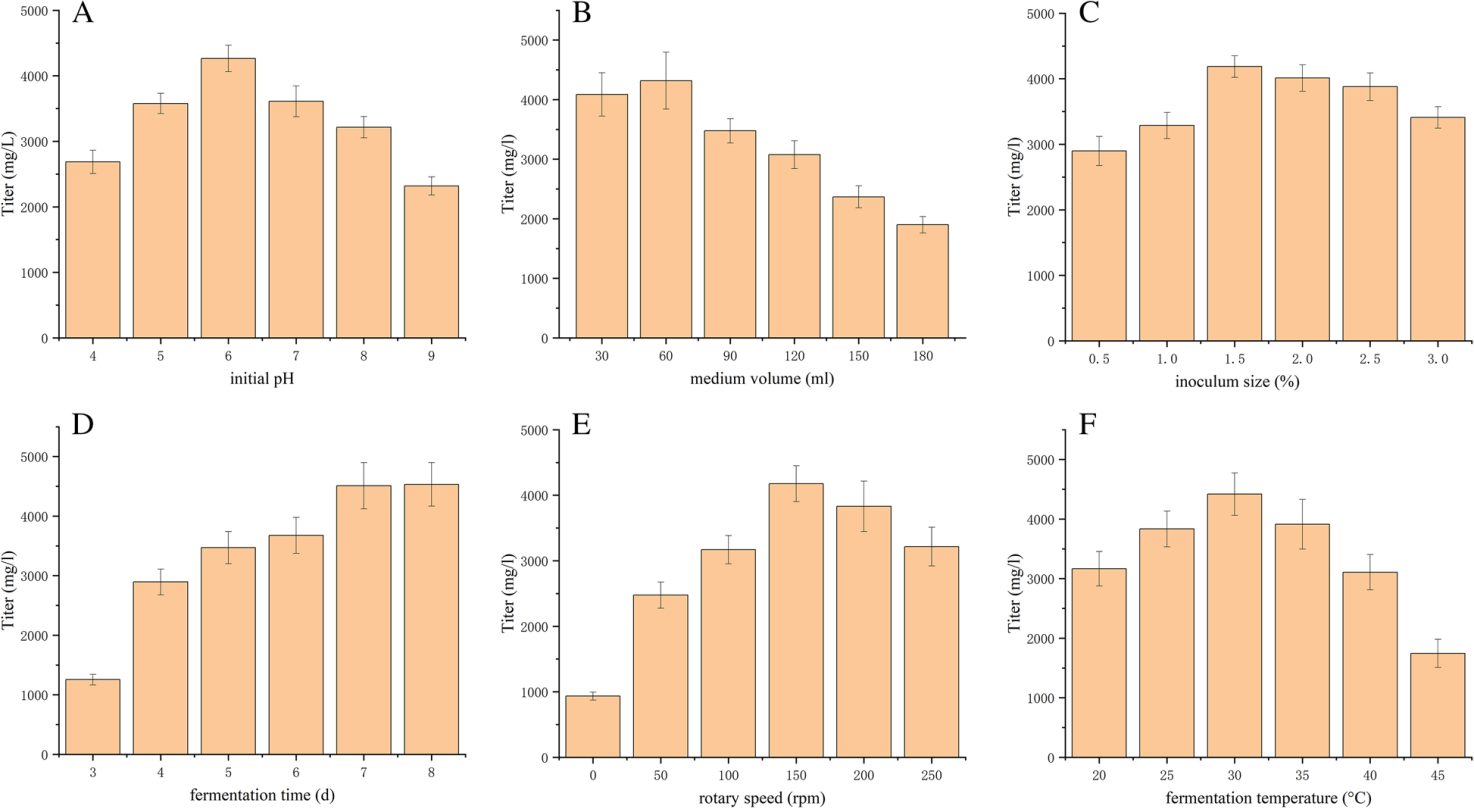
Results of one‑factor‑at‑a‑time experiments. Effect of (**A**) initial pH, (**B**) medium volume, (**C**) fermentation time, (**D**) inoculum size, (**E**) rotary speed and (**F**) fermentation temperature on antifungal substance titer

One‑factor‑at‑a‑time experiments were conducted to investigate the effect of different initial pH values, medium volumes, fermentation times, inoculum sizes, rotary speeds and fermentation temperatures on antifungal substance titers of the DS-R5 strain. Figure 5A shows the effect of medium volume in 250-ml shaking flasks on antifungal substance titers of the DS-R5 strain. The antifungal substance titer was highest when the medium volume was 50 ml, but the antifungal substance titer decreased rapidly when the medium volume was higher than 50 ml. It can be seen from Fig. 5B that the initial pH of the fermentation medium had great influence on the antifungal substance titer. When the initial pH was 4.0–9.0, the antifungal substance titer first increased and then decreased as the pH value increased. When the initial pH was 6.0, the antifungal substance titer increased significantly and reached its maximum (4322 mg/l). This indicated that the antifungal substance titer could be inhibited under conditions of excessive acidity or alkalinity. Therefore, pH 6.0 was selected as the optimal initial pH of the medium. The effect of inoculum size on the antifungal substance titer of the DS-R5 strain is shown in Fig. 5C. As inoculum size increased, the antifungal substance titer increased. At 2.0%, the antifungal substance titer reached its highest value at 4261 mg/ml. When the inoculum size was greater than 2.0%, the titer gradually decreased. Therefore, the optimal inoculum size was determined to be 2.0%. As fermentation time increased, the antifungal substance titer first increased and then tended to be stable (Fig. 5D). The titer was highest at a fermentation time of 7 days (4317 mg/ml). Therefore, the optimal fermentation time was determined to be 7 days. When the rotary speed was increased from 0 to 150 rpm, the antifungal substance titer increased. However, when the rotary speed exceeded 150 rpm, the titer began to decrease (Fig. 5E). Therefore, 150 rpm was selected as the optimal rotary speed. The effect of fermentation temperature on the antifungal substance titer of the DS-R5 strain is shown in Fig. 5F. In the range of 20–30 °C, the titer increased with increasing temperature. The titer was highest at 30 °C, then decreased rapidly at fermentation temperatures higher than 30 °C. Therefore, the fermentation temperature was set at 30 °C.

#### RSM experimental design results

##### **Optimization of culture conditions by PB design**

PB experimental design and response values are shown in Table 7. Regression analysis of each response value in Table 6 was carried out using Minitab 17.0 software to obtain the partial regression coefficient of each factor and analyze its significance. The effect size and significance of each factor after Minitab software analysis are shown in Table 8. The results of model analysis showed that the *P* value of the model was less than 0.0001, indicating that the regression equation was extremely significant. The model fit well in the entire regression region under study; the multiple correlation coefficient *R*^2^ = 0.9553 indicated good correlation, and the corrected coefficient of determination R_arj_^2^ = 0.9016 indicated that 90.16% of the variability in the experimental data could be explained by this regression model. The significance order of the six culture condition factors on the antifungal substance titer was as follows: medium volume > fermentation temperature > initial pH > inoculum size > rotary speed > fermentation time, of which medium volume, initial pH and fermentation temperature were significant factors. The regression equation derived from the above PB design was as follows: $\mathrm Y\:=\:2717-384.9\mathrm A\:+\:173.8\mathrm B-163.5\mathrm C-14.9\mathrm D\:+\:1.99\mathrm E\:+\:49.72\mathrm F$. Initial pH, rotary speed and fermentation temperature had positive effects, whereas medium volume, inoculum size and fermentation time had negative effects. Based on the above analysis, medium volume (A), initial pH (B) and fermentation temperature (F) were selected for further optimization.

**Table 7:** Design and results of PB experiments

Runs	A	B	C	D	E	F	Titer (mg/l)	
							Experimental	Predicted
1	40(-1)	7.0(+ 1)	1.5(-1)	6(-1)	120(-1)	35.0(+ 1)	4200	4237
2	60(+ 1)	5.0(-1)	1.5(-1)	6(-1)	180(+ 1)	35.0(+ 1)	3048	2986
3	40(-1)	5.0(-1)	1.5(-1)	6(-1)	120(-1)	25.0(-1)	3133	3061
4	60 (+ 1)	5.0(-1)	2.5(+ 1)	6(-1)	120(-1)	25.0(-1)	2333	2380
5	60 (+ 1)	5.0(-1)	2.5(+ 1)	8(+ 1)	120(-1)	35.0(+ 1)	2667	2699
6	40 (-1)	5.0(-1)	2.5(+ 1)	8(+ 1)	180(+ 1)	25.0(-1)	3267	3188
7	60 (+ 1)	7.0(+ 1)	1.5(-1)	8(+ 1)	120(-1)	25.0(-1)	2600	2512
8	40 (-1)	7.0(+ 1)	2.5(+ 1)	8(+ 1)	120(-1)	35.0(+ 1)	3867	3926
9	60 (+ 1)	7.0(+ 1)	2.5(+ 1)	6(-1)	180(+ 1)	35.0(+ 1)	3200	3278
10	60 (+ 1)	7.0(+ 1)	1.5(-1)	8(+ 1)	180(+ 1)	25.0(-1)	3000	2943
11	40 (-1)	5.0(-1)	1.5(-1)	8(+ 1)	180(+ 1)	35.0(+ 1)	3667	3602
12	40 (-1)	7.0(+ 1)	2.5(+ 1)	6(-1)	180(+ 1)	25.0(-1)	3333	3302

**Table 8:** Analysis of data generated using PB design

Coded variable	Variable	Coefficient estimate	*T*-value	*P*-value	Significance
Intercept	Constant	3192.9	65.93	0.000	***
A	medium volume	-384.9	-7.85	0.001	***
B	initial pH	173.8	3.59	0.016	*
C	inoculum size	-81.8	-1.69	0.152	
D	fermentation time	-14.9	-0.31	0.771	
E	rotary speed	59.6	1.23	0.273	
F	fermentation temperature	248.6	5.13	0.004	**

*Note:*

^*^Significant at the *P* < 0.05 level

^**^Significant at the *P* < 0.01 level

^***^ Significant at the *P* < 0.001 level

##### **Steepest ascent design**

The coefficient estimates for medium volume, initial pH and fermentation temperature presented in Table 8 indicated that initial pH and fermentation temperature had positive effects, whereas medium volume had negative effects on the titer of antifungal substance. Based on the above results, the directions of the gradients for medium volume, initial pH and fermentation temperature were determined using steepest ascent design (Table 9). As shown in Table 9, the path of steepest ascent started from the center of the variables chosen from the PB design and moved along the path, with medium volume, initial pH and fermentation temperature moving by 5, 0.5 and 1.0, respectively. The maximum titer was obtained in test 3, when the point was nearest the maximum titer response. Therefore, this point (medium volume 50 ml, initial pH 7.0, fermentation temperature 32 °C) was chosen as the center point for further optimization by CCD.

**Table 9:** Steepest ascent path experimental design and results

Test	medium volume (ml)	initial pH	fermentation temperature (°C)	Titer (mg/l)
1	60	6.0	30.0	6363
2	55	6.5	31.0	6889
3	50	7.0	32.0	7467
4	45	7.5	33.0	7148
5	40	8.0	34.0	6422
6	35	8.5	35.0	5012
7	30	9.0	36.0	4566

##### **Optimization of significant variables using CCD**

To find the optimal culture conditions for antifungal substance production, CCD with five coded levels was used to further optimize conditions with regard to medium volume, initial pH and fermentation temperature. The experimental design and response values from CCD are shown in Table 10. A quadratic regression equation with the titer of antifungal substance as the objective function was established, and variance analysis and significance tests were performed on the obtained regression equation. After regression analysis of the experimental data, the quadratic polynomial equation was obtained as follows:

**Table 10:** Experimental design and response values from CCD

Run	Factors			Titer (mg/l)	
	A:medium volume (ml)	B:initial pH	F:fermentation temperature (°C)	Experimantal	Predicted
1	+ 1.682(58)	0(7.0)	0(32)	6199	6235
2	0(50)	0(7.0)	0(32)	7470	7398
3	+ 1(55)	-1(6.5)	+ 1(33)	5990	6026
4	0(50)	0(7.0)	0(32)	7691	7639
5	+ 1(55)	-1(6.5)	-1(31)	5782	5814
6	-1(45)	-1(6.5)	-1(31)	5733	5688
7	-1.682(42)	0(7.0)	0(32)	5720	5673
8	0(50)	-1.682(6.2)	0(32)	6132	6185
9	0(50)	0(7.0)	-1.682(30.3)	5619	5725
10	+ 1(55)	+ 1(7.5)	-1(31)	6353	6279
11	-1(45)	+ 1(7.5)	-1(31)	5561	5607
12	+ 1(55)	+ 1(7.5)	+ 1(33)	6899	6823
13	-1(45)	+ 1(7.5)	+ 1(33)	5886	5936
14	0(50)	+ 1.682(7.8)	0(32)	6341	6281
15	0(50)	0(7.0)	+ 1.682(33.7)	6058	6090
16	0(50)	0(7.0)	0(32)	7900	7983
17	0(50)	0(7.0)	0(32)	7863	7793
18	-1(45)	-1(6.5)	+ 1(33)	5659	5588
19	0(50)	0(7.0)	0(32)	7893	7953
20	0(50)	0(7.0)	0(32)	7937	8025

$$\mathrm Y\:=\:-771949\:+\:1668\;\mathrm A\:+\:20683\;\mathrm B\:+\:41347\;\mathrm F-25.25\;\mathrm A^2-2133\;\mathrm B^2-674.0\;\mathrm F^2\:+\:71.2\;\mathrm{AB}\:+\:12.6\;\mathrm{AF}\:+\:184\;\mathrm{BF},$$

where Y is the titer of antifungal substances and A, B and F are the coded factors medium volume, initial pH and fermentation temperature, respectively. Standard variance analysis of the regression model is shown in Table 11. The regression model had a *P* value < 0.0001, revealing that the model was statistically significant. The lack of fit was not significant, indicating that any inability to fit caused by experimental error could be ignored. The model terms A, B, F, AB, A^2^, B^2^ and F^2^ were found to be significant, which revealed that medium volume, initial pH, fermentation temperature and the interaction of medium volume with initial pH were significant. The correlation coefficient of the regression equation was *R*^2^ = 0.9810, indicating that the obtained equation had good fit and that this equation could be used to predict test results.

**Table 11:** ANOVA analysis for CCD regression equations

Source	Sum of squares	Df	Mean square	*F-*value	*P*-value
Model	15206097	9	1689566	57.29	0.000***
A	654877	1	654877	22.21	0.001**
B	260592	1	260592	8.84	0.014*
F	222534	1	222534	7.55	0.021*
A^2^	5742543	1	5742543	194.73	0.000***
B^2^	4098925	1	4098925	139.00	0.000***
F^2^	6547266	1	6547266	222.02	0.000***
AB	253828	1	253828	8.61	0.015*
AF	31626	1	31626	1.07	0.325
BF	67896	1	67896	2.30	0.160
Residual	294893	10	29489		
Lack of Fit	133078	5	26616	0.82	0.582
Pure error	161815	5	32363		
Cor total	1915500990	19			

*R*^2^ = 0.9810, adjusted *R*^2^ = 0.9639, predicted *R*^2^ = 0.9186

*Note:*

^*^Significant at the *P* < 0.05 level

^**^Significant at the *P* < 0.01 level

^***^ Significant at the *P* < 0.001 level

**Fig. 6:**
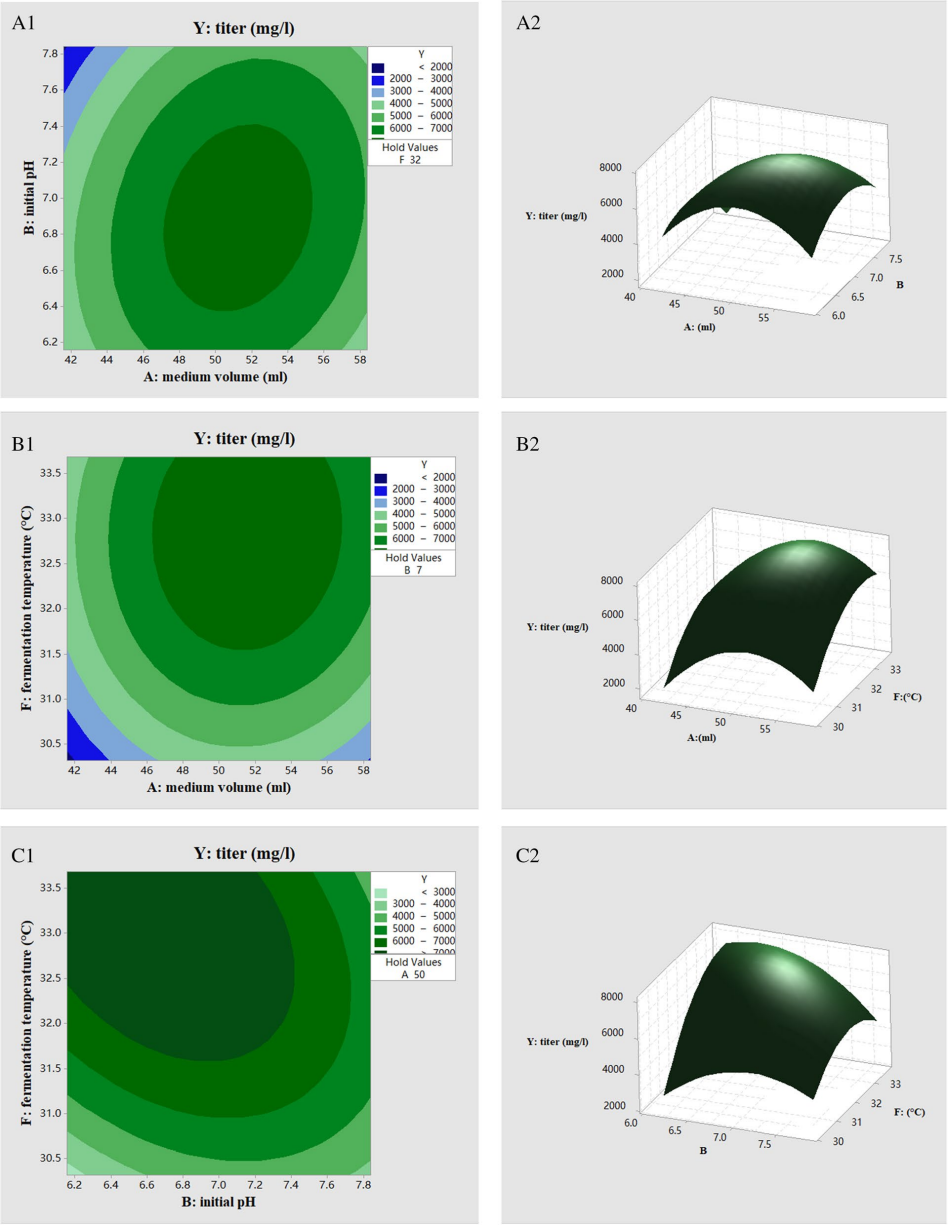
Response surface curve and corresponding contour plot of the effect of three variables on antifungal substance titer. **A** interaction of medium volume and initial pH; **B** interaction of medium volume and fermentation temperature; **C** interaction of initial pH and fermentation temperature

According to the obtained quadratic regression equation and variance analysis results, the contour plot and corresponding response surface curve was drawn using Minitab software to more intuitively observe the effect of the interactions between factors on the titer of antifungal substance (Fig. 6). When one factor was fixed at the optimal value, the titer of antifungal substance first increased then decreased as the other two factors increased until the vertex of the curved surface reached the maximum titer point. In the contour plot map, closer circles indicate less significant interactions between the two factors, whereas an ellipse represents a significant interaction [25]. The contour plot maps showed that the response surface curve of the interaction between medium volume and initial pH was the most curved, indicating that the interaction between these two factors was stronger than the interactions between other factors, which was also consistent with the results of variance analysis. In order to determine the optimal values of the main influencing culture condition factors, the derivation of three independent variables of the regression equation was obtained. With the derivative equal to 0, the extreme point of the model was obtained as follows: medium volume, 51.0 ml; initial pH, 6.7; fermentation temperature, 33.1 °C. According to PB design results, the optimal values of other culture conditions were as follows: inoculum size, 1.5%; fermentation time, 6.0 days; rotary speed, 180 rpm. Under these optimal conditions, the model predicted a maximum antifungal substance titer of 8036 mg/l, which was a 77.6% increase compared to the original culture conditions (4357 mg/l). In order to verify the feasibility of RSM, the optimal culture conditions obtained by regression analysis were verified. The validation test was repeated three times, and the average value was 4295 mg/l, which was not significantly different from the predicted value. The experimental results showing that the validation value was close to the predicted value indicated that the model reflects the actual fermentation situation and can be used to predict the relationship between the antifungal substance titer and fermentation conditions.

## Discussion

Although 16S rRNA gene sequences are widely used to identify bacteria and construct phylogenetic relationships between bacteria, most 16S rRNA gene-based phylogenetic tree topologies are not reliable due to high similarity between sequences among taxa with close kinship [26]. In recent years, it has been found that many protein-coding genes can also be used as markers for phylogenetic identification. These include the *gyrA*, *gyrB* and *ropD* genes, and their use can compensate for the deficiencies of the 16S rRNA gene [27]. Among these protein-coding genes, the molecular evolution rate of the *gyrB* gene is higher than that of the 16S rRNA gene, so it is often used for the identification of closely related species [28]. In this study, the DS-R5 strain was identified as *P*. *polymyxa* by colony morphology and cellular, physiological and biochemical characteristics combined with 16S rRNA and *gyrB* gene sequence analysis.

**Fig. 7:**
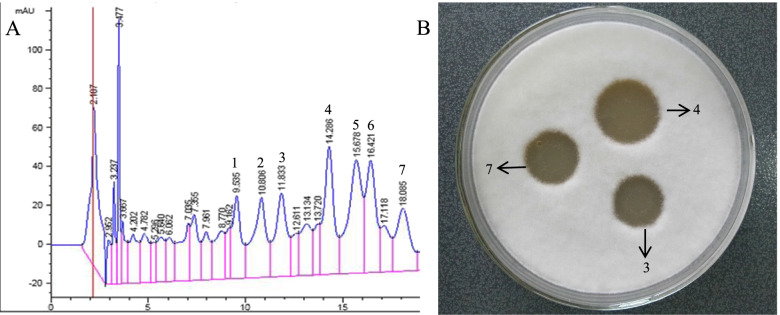
The HPLC and the antifungal activity result of the isolated substances

In our previous stuy, the antifungal substances produced by the DS-R5 strain were purified by using hydrochloric acid precipitation, methanol extraction and molecular sieve, and then freeze-dried to obtain the crude active substances. The crude is further separated by high performance liquid chromatography (HPLC) under the following conditions: Ultimate XB-C18 semi-preparative column (21.2 × 250 mm, 5 µm), flow rate 5.0 ml/min, column temperature 25 °C, injection volume 1 ml, mobile phase ratio: acetonitrile: water (55:45, adding 0.1% trifluoroacetic acid). The results of HPLC showed that seven main substances were isolated from the fermentation broth of the DS-R5 strain (Fig. 7A). Antifungal activity tests revealed that three of these seven substances had antifungal activity (Fig. 7B). These experiments also showed that the yields of the three antifungal substances were not all proportional to the results of culture condition optimization. Thus, we could not use the yield of one of the antifungal substances as the response variable for optimizing culture conditions. Therefore, when we carried out RSM to optimize the culture conditions, we selected the total antifungal effect of these three antifungal substances as the response variable. Generally speaking, the evaluation of the antimicrobial effect of biocontrol strains is based on the amount of a specific antimicrobial substance or the width of the inhibition zone. Zhao et al. optimized the fermentation conditions of the *Bacillus* sp. BH072 strain by RSM using the amount of antifungal substance iturin A as the optimization response variable [29]. Ju et al. optimized the nutritional requirements for antimicrobial activity of *Streptomyces rimosus* AG-P1441 using the width of the inhibition zone as optimization response variable [30]. In this study, we use the titer of the fermentation broth of the DS-R5 strain as the optimization response variable. Since the titer of the fermentation broth is proportional to the diameter of the inhibition zone, in essence, we also use the the diameter of inhibition zone as the response variable in the optimization process.

The production of antifungal substances by many biocontrol bacteria is generally believed to be an aerobic process [31], so dissolved oxygen is an important factor in the fermentation of *P. polymyxa*. In shake flasks, oxygen supply is related to the volume of media in the flask and the rotary speed. Decreasing the medium volume or increasing the rotary speed can improve dissolved oxygen levels in the medium. However, too little media or too high rotary speeds are also not conducive to the production of antifungal substances. If the amount of medium in the flask is too small, increased evaporation of the solution during the fermentation process can lead to large errors in the results. Similarly, once the rotary speed of the shaker reaches a certain value, the amount of dissolved oxygen will not increase further because of the upper limit of the dissolved oxygen concentration in water. In this study, medium volume was found to be the most important variable influencing the titer of antifungal substances of *P. polymyxa* DS-R5 at an individual level and showed significant influence at an interactive level. The titer of antifungal substances increased as medium volume in the flask decreased, indicating that higher dissolved oxygen levels are beneficial to antifungal substance production. The rotary speed of the shaker had a positive effect on the titer of antifungal substances, indicating that an appropriate increase in rotary speed would increase the titer of antifungal substances. However, the effect of rotary speed on the titer of antifungal substances was not significant, indicating that the range of rotary speeds we selected in PB design experiments had reached the upper limit and could not be further increased.

*P. polymyxa* can produce a variety of antifungal substances, such as peptides, proteins, nucleosides, pyrazines and phenols, and can prevent and control a variety of plant diseases [13]. The production of *Bacillus* secondary substances is affected by the composition of culture medium and fermentation conditions. Different medium components and fermentation conditions can significantly affect the growth of strains and the production of metabolites. There have been many previous RSM optimization studies on the antagonistic effect of antifungal substances. Zhao *et al*. demonstrated that the antifungal apecific activity of the *Bacillus* sp. BH072 strain was enhanced from 350.11 AU/mg to 513.92 AU/mg under the optimized medium and culture conditions, which was 46.8% higher than the previous result [29]. Wang *et al*. optimized the culture conditions of the *P. polymyxa* Cp-S316 strain by using ractional factorial design and central composite designs. Anfungal substances production could reach 4687.71 μg/ml, which was 3.05 times higher than that without optimization [32]. In this study, the antifungal substances production can reach 8036 mg/l after optimization by one‑factor‑at‑a‑time experimental design and CCD, which was a 77.6% increase compared to the original culture conditions. The optimization results indicate that RSM can be well used to optimize the culture conditions for antifungal substance and provides an important experimental basis for the industrial-scale production of the DS-R5 strain.

**Fig. 8:**
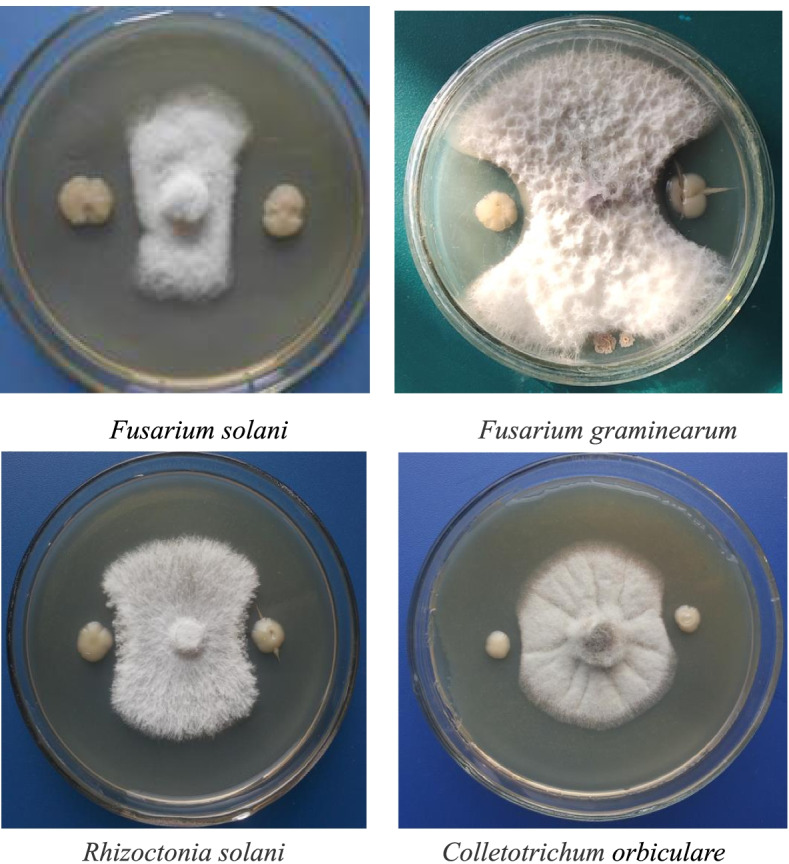
Antagonistic effect of DS-R5 strain on some pathogenic fungi

This study also found that both live DS-R5 bacteria and the antifungal substances produced in the fermentation broth could inhibit the growth of *F. solani* (Fig. 8), thereby allowing for biological control of *S. miltiorrhiza* root rot. Regarding the future application and development of *S. miltiorrhiza* root rot control measures, we recommend the use of live bacteria because the antifungal substances extraction process is cumbersome. In addition, the application of spores produced by the DS-R5 strain is also recommended because isolation of antifungal substances produced by the DS-R5 strain can be performed rapidly. This study isolating *P. polymyxa* DS-R5 from *S. miltiorrhiza* lays a strong foundation for the biological control of *S. miltiorrhiza* root rot and provides a basis for future studies clarifying the effect of *P. polymyxa* DS-R5 on *S. miltiorrhiza* root rot. This study provides theoretical guidance for the development and application of biocontrol strains for *S. miltiorrhiza* root rot and provides functional strains and effective metabolites for the development of new drugs.

## Conclusion

In this work we studied the isolation and screening of biocontrol bacteria strains that prevent and control *S. miltiorrhiza* root rot. A total of 72 strains of bacteria were isolated from the roots, stems and leaves of *S. miltiorrhiza*. Among the 72 isolates obtained from root, stem and leaf tissue, the DS-R5 strain had the strongest inhibitory effect against the target pathogenic fungus *F. solani*. The DS-R5 strain was identified as *P*. *polymyxa* by colony morphology and cellular, physiological and biochemical characteristics combined with 16S rRNA and *gyrB* gene sequence analysis. In addition, using one‑factor‑at‑a‑time experimental design, PB design and CCD, the fermentation conditions for production of antifungal substances by *P*. *polymyxa* DS-R5 in a shake flask system were optimized. The optimized fermentation conditions were as follows: medium volume, 51.0 ml; initial pH, 6.7; fermentation temperature, 33.1 °C; inoculum size, 1.5%; fermentation time, 6.0 days and rotary speed 180 rpm. Significantly higher antifungal substance titers (8036 mg/l) were obtained under the optimized fermentation conditions than under original fermentation conditions. To the best of our knowledge, this study is the first to isolate and purify biocontrol bacteria that prevent and control *S. miltiorrhiza* root rot and to optimize culture conditions for biocontrol bacteria to produce antifungal substances using PB design and CCD experiments.

## Data Availability

All data and material are available upon request to correspondence author.
